# Synthesis of L-Ascorbyl Flurbiprofenate by Lipase-Catalyzed Esterification and Transesterification Reactions

**DOI:** 10.1155/2017/5751262

**Published:** 2017-03-21

**Authors:** Jia-ying Xin, Li-rui Sun, Shu-ming Chen, Yan Wang, Chun-gu Xia

**Affiliations:** ^1^Key Laboratory for Food Science & Engineering, Harbin University of Commerce, Harbin 150076, China; ^2^State Key Laboratory for Oxo Synthesis & Selective Oxidation, Lanzhou Institute of Chemical Physics, Chinese Academy of Sciences, Lanzhou 730000, China; ^3^College of Animal Science and Veterinary Medicine, Shanxi Agricultural University, Taigu 030801, China

## Abstract

The synthesis of L-ascorbyl flurbiprofenate was achieved by esterification and transesterification in nonaqueous organic medium with Novozym 435 lipase as biocatalyst. The conversion was greatly influenced by the kinds of organic solvents, speed of agitation, catalyst loading amount, reaction time, and molar ratio of acyl donor to L-ascorbic acid. A series of solvents were investigated, and tert-butanol was found to be the most suitable from the standpoint of the substrate solubility and the conversion for both the esterification and transesterification. When flurbiprofen was used as acyl donor, 61.0% of L-ascorbic acid was converted against 46.4% in the presence of flurbiprofen methyl ester. The optimal conversion of L-ascorbic acid was obtained when the initial molar ratio of acyl donor to ascorbic acid was 5 : 1. kinetics parameters were solved by Lineweaver-Burk equation under nonsubstrate inhibition condition. Since transesterification has lower conversion, from the standpoint of productivity and the amount of steps required, esterification is a better method compared to transesterification.

## 1. Introduction

The treatment of Alzheimer's disease is still a major challenge for the medical field. The clinical failure of efficient Alzheimer's disease drug delivery may be largely attributed to the low permeability of drugs due to the blood-brain barrier and a lack of appropriate drug delivery systems [1]. Flurbiprofen is one of the most potent nonsteroidal anti-inflammatory drugs (NSAIDs) and may help to prevent Alzheimer's disease [2, 3]. However, poor brain delivery of flurbiprofen and its serious gastrointestinal side effects have hampered the application of flurbiprofen as neuroprotective agents [4].

Localized and controlled delivery of drugs at their desired site of action can reduce toxicity and increase treatment efficiency. L-Ascorbic acid is essential for many enzymatic reactions, which is transported directly across the blood-brain barrier via Naþ-dependent vitamin C transporter SVCT2 and particularly prevalent in the brain [1]. It has been reported that L-ascorbic acid could be used as a carrier to promote brain drug delivery [5–7]. To overcome the problems of the low blood-brain barrier permeability of flurbiprofen and to increase its delivery to the brain for the treatment of Alzheimer's disease, one attractive approach is to design and synthesize flurbiprofen ester prodrug named L-ascorbyl flurbiprofenate containing ascorbate as a specific carrier system for brain delivery.

So far, there are very few reports on the lipase-catalyzed synthesis of L-ascorbyl flurbiprofenate. Wang and Tang studied the synthesis of L-ascorbyl flurbiprofenate by lipase-catalyzed esterification of L-ascorbic acid with flurbiprofen in tertiary amyl alcohol but no optimum operating parameters were provided [8]. Liu and Tang investigated the kinetics and thermodynamics of the lipase-catalyzed esterification of L-ascorbic acid with flurbiprofen in 2-methy-l,2-butanol [9]. Only limited information on the lipase-catalyzed esterification was available. There were no reports on the lipase-catalyzed synthesis of L-ascorbyl flurbiprofenate by transesterification. However, solvent properties, quantity of enzyme, and molar ratio of L-ascorbic acid to flurbiprofen may influence the biocatalytic reaction. So far, the influence of these factors for the synthesis of L-ascorbyl flurbiprofenate has not been investigated in detail. To obtain high conversion, it is important to determine the optimal reaction conditions and understand the kinetics parameters.

The present study focused on lipase-catalyzed synthesis of L-ascorbyl flurbiprofenate. The lipase-catalyzed esterification and transesterification approaches have been compared. It was worthwhile to compare the merits and demerits of the two processes and optimize process conditions.

## 2. Experimental

### 2.1. Materials

Novozym 435 lipase (Lipase B from* Candida antarctica* immobilized on macroporous acrylic resin; specific activity: 10,000 U/g) was purchased from Novozymes, Denmark. Porcine pancreas lipase Type II (powder, 30–90 U/mg) and* Candida rugosa* lipase (Type VII, powder, 706 U/mg) were purchased from Sigma.

(*R*, *S*)-flurbiprofen (purity > 99%) was purchased from Shanghai Mei Lan Chemical Co. Ltd. (Shanghai, China). The purity of substrates is over 99.7% for L-ascorbic acid. All solvents were dehydrated before use with activated 3 Å molecular sieves. Thin-layer chromatography (TLC) plates were purchased from Merck (KGaA, Darmstadt, Germany).

### 2.2. Synthesis of Ester

The methyl ester of (*R*, *S*)-flurbiprofen was prepared by the classical methodology using thionyl chloride and methanol. Thionyl chloride, 15 mL (0.20 mol), was added dropwise to cooled, stirred suspension of (*R*, *S*)-flurbiprofen (0.12 mol) in methanol (250 mL). The reaction mixture was refluxed for 2.5 h, and then the solvent was evaporated and the residue purified by column chromatography using SiO_2_ as adsorbent and petroleum ether purified as eluant and its purity was > 98% using the HPLC methods described in a later section.

### 2.3. Reaction Conditions

Unless otherwise stated, the esterification and transesterification reactions were performed in 50 mL closed, screw-capped glass vials containing 25 mL of organic solvent, flurbiprofen or flurbiprofen methyl ester (26–260 mmol/L), L-ascorbic acid (26 mmol/L), and 800 mg of molecular sieve 3 Å. The reaction had been started by adding 40 mg of lipase. The headspace in the vials was filled with nitrogen gas and refilled after each sampling. The reaction mixture was stirred with a magnetic stirrer at different temperature. In both cases, samples were withdrawn at specified time intervals for measurement of the conversion. Control experiments without enzymes were carried out in parallel.

### 2.4. Analytical Methods

In order to monitor the reaction progress, samples of 100 *μ*L were withdrawn at intervals and analyzed by thin-layer chromatography (TLC) on silica gel 60 F_254_ plates with fluorescent indicators. The TLC migration was carried out with a solvent mixture of chloroform/methanol/glacial acetic acid/water (80 : 10 : 8 : 2, V/V/V/V). The TLC plates were visualized under UV. Results were estimated from intensity of spots on TLC.

Quantitative analysis was done by HPLC (Thermo U3000), on a XDB C18 reversed phase column (5 *μ*m, 4.6 × 150 mm) with acetonitrile/water/formic acid (80/20/0.2, V/V/V) as mobile phase at 1 mL/min flow rate. Detection was achieved using UV detection at 294 nm. Samples were filtered to remove the enzyme and molecular sieves and 100 *μ*L of the solution was diluted with acetonitrile/water/formic acid (80/20/0.2, V/V/V). L-Ascorbic acid was a limiting substrate throughout this study; therefore, the conversion was calculated as the ratio in moles of the product to initial L-ascorbic acid. All experiments were performed in triplicate and standard deviations were calculated.

The enantiomeric excess values of flurbiprofen and flurbiprofen methyl ester (ee) were determined by HPLC (Agilent 1200) by using a chiral column (Chiralcel OD-H, 5 *μ*m, 250 mm × 4.6 mm) capable of separating the *R*- and *S*-isomers of flurbiprofen and flurbiprofen methyl ester, and the mobile phase was hexane/isopropanol/trifluoroacetic acid solution (98 : 2 : 0.1, V/V/V), at a flow rate of 1.0 mL/min. UV detection at 294 nm was used for quantification at the 25°C. The enantiomeric excess (ee) was obtained from peak areas of the *R*- and *S*-isomers of flurbiprofen or flurbiprofen methyl ester by the following equation: ee = (*A*_*S*_ − *A*_*R*_)/(*A*_*S*_ + *A*_*R*_), where *A*_*S*_ is peak areas of the *S*-isomer and *A*_*R*_ is peak areas of the *R*-isomer.

### 2.5. Kinetic Study

Reactions were carried out no more than 5% conversion and the initial rate was determined as the slope of the reaction curve tangent to the initial stage of the reaction and expressed as mol of products h^−1^ L^−1^. Because all experiments were performed in triplicate, reaction curves were constructed using average values of the reaction rate for each experimental point. A linear portion of the reaction curve at various substrate concentrations consisted of 5 experimental points, where the number of experimental points included was determined by the condition that correlation coefficients of the initial straight line must be above 0.95.

## 3. Results and Discussion

### 3.1. Esterification and Transesterification

**Figure 1:**
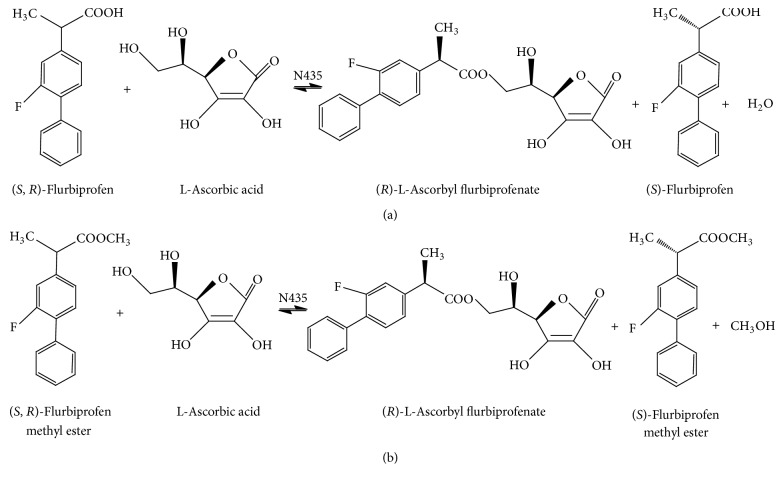
Novozym 435 lipase-catalyzed esterification (a) and transesterification (b).

It was previously reported that esterification of flurbiprofen with L-ascorbic acid by lipase in tert-amyl alcohol produced L-ascorbyl flurbiprofenate regioselectively, which was only the product identified in the HPLC analysis [9]. In this paper, lipase-catalyzed acylation of L-ascorbic acid was performed in presence of racemic flurbiprofen (esterification) or racemic flurbiprofen methyl ester (transesterification). Both kinds of reaction led to only one product identified as L-ascorbyl flurbiprofenate in the HPLC analysis. Furthermore, the chiral HPLC analysis indicated that the lipases also display stereospecificity in the esterification and transesterification. The *R*-isomer reacted with the Novozym 435 lipase; the *S*-isomer reacted with the* Candida rugosa* lipase and porcine pancreas lipase cannot catalyze the reaction. It has been reported that *S*-flurbiprofen and *R*-flurbiprofen have the same physiological activity in prevention of the development of Alzheimer's disease but *R*-flurbiprofen has reduced side effects related to inhibition of cyclooxygenase (COX) [10–12]. Thus, Novozym 435 lipase, identified as a *R*-stereospecific catalyst, has been employed in further experiments. The two reactions for the preparation of L-ascorbyl flurbiprofenate are shown in Figure 1.

### 3.2. Effect of Solvent

Different organic solvents had different ability to distort the essential water layer around lipase and could greatly influence the activity of lipase. Moreover, organic solvent relatively influenced the solubility of the substrates, and thus it would affect the synthesis of the L-ascorbyl flurbiprofenate. The polarity of L-ascorbic acid is very different from those of flurbiprofen and flurbiprofen methyl ester. Because of this significant difference in polarity, such a solvent with relatively high solubility of flurbiprofen, flurbiprofen methyl ester, and L-ascorbic acid needs to be found.

Therefore, esterification and transesterification strategies for the lipase-catalyzed synthesis of L-ascorbyl flurbiprofenate were compared using different organic solvents. The effect of various organic solvents on conversion of L-ascorbic acid was studied under similar conditions using 50 mg Novozym 435 lipase, 800 mg of molecular sieve 3 Å, 2.3 mmoles of flurbiprofen or flurbiprofen methyl ester, 0.57 mmoles of ascorbic acid, and 25 mL organic solvent at 160 rpm, at 50°C for 72 h. It was clear from Table 1 that the type of organic solvent strongly influenced the synthesis of L-ascorbyl flurbiprofenate. In the case of esterification and transesterification, tert-butanol was the best solvent with tert-amyl alcohol as the next best. However, the reaction product was not inspected in benzene and toluene.

log⁡*P* is widely used to represent the characteristics of the organic solvent system where *P* is the partition coefficient of the solvent between water and octanol [13]. It is generally reported that solvents with log⁡*P* < 2 are high polarity that may strip water from enzyme molecules easily and are less suitable for biocatalytic purpose [14]. In this case, no correlation of the lipase activity with log⁡*P* of the solvents could be established. This could be attributed that L-ascorbic acid had very low concentration in solvents with high log⁡*P*. Since tert-butanol was the optimum solvent for esterification and transesterification, it was used as the solvent for transesterification and esterification in further experiments.

### 3.3. Effect of Speed of Agitation

**Figure 2:**
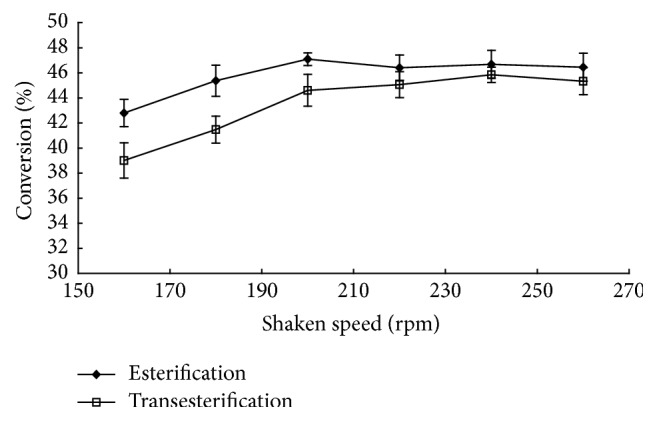
The effect of speed of agitation on the conversion of esterification and transesterification.

In the case of immobilized enzyme, external mass transfer limitations may be important. The reactants have to diffuse from the bulk liquid to the external surface of the catalyst. External mass transfer can be minimized by carrying out the reaction at an optimum speed of agitation. The effect of speed of agitation was studied both for esterification and transesterification over the range of 160–260 rpm by taking 50 mg Novozym 435 lipase, 800 mg of molecular sieve 3 Å, 2.3 mmoles of flurbiprofen or flurbiprofen methyl ester, 0.57 mmoles of ascorbic, and 25 mL tert-butanol at 50°C for 72 h. The conversions in both the cases were independent of the speed of agitation at and beyond 180 rpm for esterification and transesterification (Figure 2). This indicated that the influence of external mass transfer limitation was negligible and a speed of agitation of 180 rpm did not limit reaction rate. So all subsequent experiments were carried out at 180 rpm.

### 3.4. Effect of Catalyst Loading Amount

**Figure 3:**
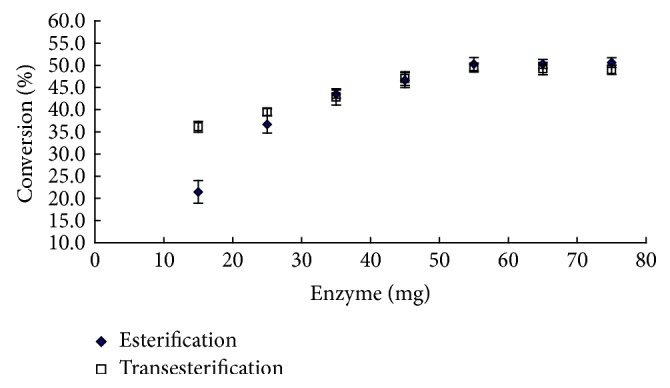
The effect of catalyst loading amount on the conversion of esterification and transesterification.

Internal diffusion problems could happen when the substrate could not reach the inner parts of the support. L-Ascorbyl flurbiprofenate synthesis as a function of catalyst loading amount was studied at 50°C and 180 rpm for 72 h. The conversion of the reaction increased with increasing immobilized lipase loading ranging from 15 mg to 55 mg in both the cases; a linear relationship between the conversion and enzyme load demonstrated that the internal diffusion limitations could be minimized. Maximal conversion was achieved with 55 mg lipase for esterification and transesterification after conversion became constant and no further increases (Figure 3).

Also, in the case of esterification reaction, the conversion was lower than the transesterification reaction under 15 mg and 25 mg lipase addition and the asymptotes were seen in the conversion for transesterification and esterification when the amount of enzyme added was beyond 35 mg. Since there was no significant increase in the conversion with increased catalyst loading from 55 mg to 75 mg, further parameters were studied using 55 mg catalyst loading.

### 3.5. Effect of the Molar Ratio of Acyl Donor to Ascorbic Acid

**Figure 4:**
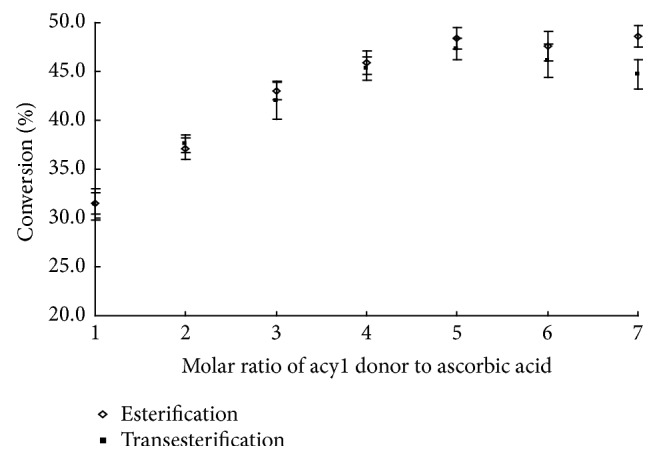
The effect of the molar ratio of acyl donor to ascorbic acid on the conversion of esterification and transesterification.

The molar ratio of one substrate to another is an important parameter affecting the conversion. The solubility of L-ascorbic acid in tert-butanol at 50°C was 26 mmol/L, which is saturated in tert-butanol that would be limiting as substrate. Therefore, the effect of the molar ratio of the reactants was studied by keeping the concentration of L-ascorbic acid and the catalyst quantity constant and varying the concentration of acyl donor in both esterification and transesterification.  Figure 4 showed the effect of the molar ratio on the conversion for the synthesis of L-ascorbyl flurbiprofenate. Molar ratio of acyl donor to ascorbic acid was varied from 1 : 1 to 7 : 1, and the L-ascorbic acid was kept constant. The conversion at a reaction time of 72 h for esterification and transesterification was compared. Indeed, within experimental error, at a fixed concentration of L-ascorbic acid and loading amount of Novozym 435 lipase, the conversion increased with increasing acyl donor concentrations up to a critical value. For esterification and transesterification, when using higher ratios of flurbiprofen or flurbiprofen methyl ester over L-ascorbic acid, conversion increased and highest conversion (61.0% for esterification and 46.4% for transesterification) occurred at 5 : 1 molar ratio of flurbiprofen or flurbiprofen methyl ester to L-ascorbic acid. At molar ratios greater than 5 : 1, a constant conversion was observed.

### 3.6. Effect of Reaction Time

**Figure 5:**
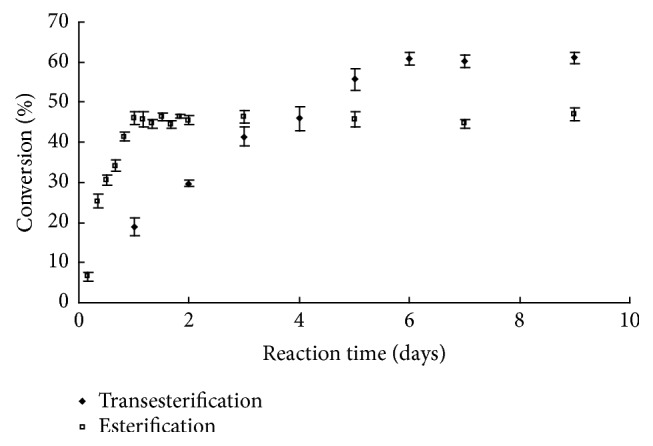
Time course of the synthesis of L-ascorbyl flurbiprofenate.

Figure 5 showed the changes in the conversion with time for the direct esterification and transesterification in tert-butanol at 50°C with molar ratio of acyl donor to ascorbic acid of 5 : 1. Direct esterification and transesterification reaction had a different initial rate and maximal conversion. In the case of transesterification reaction, the rate was faster than that of the esterification reaction under otherwise similar conditions within the reaction time of 24 h. This could be attributed that lipase had higher activity for flurbiprofen methyl ester and lower activity with flurbiprofen. Also the difference consisted in the maximal conversion of L-ascorbic acid, which was 61.0% occurring after 144 h of incubation for esterification, against 46.4% occurring after 72 h of incubation for transesterification. This difference might be due to methanol production during the transesterification reaction which was not eliminated by the 3 Å molecular sieve and disadvantageous reaction equilibrium for L-ascorbyl flurbiprofenate production. Even if water was also produced as a coproduct during the esterification reaction, the 3 Å molecular sieve favors its elimination by adsorption and then contributes to shift the reaction equilibrium towards the synthesis of L-ascorbyl flurbiprofenate.

### 3.7. Kinetic Study

**Figure 6:**
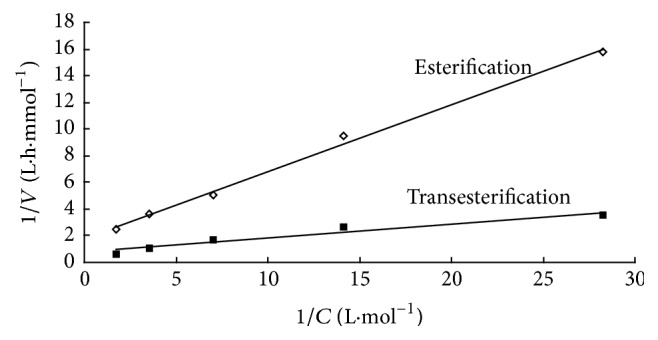
The Lineweaver-Burk plot of 1/*V*_0_ versus 1/[*S*].

The effects of several parameters including organic solvent, speed of agitation, enzyme amount, substrate molar ratio, and reaction time on conversion of L-ascorbic acid were investigated to optimize the conditions. Since L-ascorbic acid was dissolved in tert-butanol with a saturated concentration of 26 mmol/L, the effect of concentration of flurbiprofen or flurbiprofen methyl ester on the rate of reaction was investigated systematically over a wide range with a constant L-ascorbic acid concentration. For the determination of initial rates, esterification and transesterification were conducted by using 55 mg Novozym 435 lipase with appropriate quantities of acyl donor (flurbiprofen or flurbiprofen methyl ester) and L-ascorbic acid. In esterification and transesterification experiments, the amount of flurbiprofen was varied from 35 to 568 mmol/L at a fixed quantity of L-ascorbic acid (26 mmol/L). The initial rates were determined for each experiment. From the initial rate determined, it showed that when the concentration of acyl donor (flurbiprofen or flurbiprofen methyl ester) was increased, by keeping the concentration of L-ascorbic acid constant, the initial rate of reaction increased proportionally. This presumed that there was no evidence of inhibition by acyl donor (flurbiprofen or flurbiprofen methyl ester) at all the concentrations tested. According to the rate equation for the ping-pong bi-bi mechanism without substrate inhibition [15], the Lineweaver-Burk plot of 1/*V*_0_ versus 1/[*S*] in Figure 6 showed the kinetic parameter values. The apparent Michaelis-Menten kinetics are *K*_rn_ = 0.29 mol/L and apparent *V*_max_ = 0.563 mmol/L·h for flurbiprofen. Apparent Michaelis-Menten kinetics are *K*_rn_ = 0.14 mol/L and apparent *V*_max_ = 1.34 mmol/L·h for flurbiprofen methyl ester. This indicated that Novozym 435 lipase has higher affinity for flurbiprofen methyl ester and lower affinity with flurbiprofen. Transesterification has higher reaction rate than esterification.

## 4. Conclusion

The L-ascorbyl flurbiprofenate has been synthesized successfully by lipase-catalyzed transesterification and esterification in tert-butanol. The goal of this work was to compare lipase-catalyzed esterification and transesterification approaches. Both approaches were studied in a systematic way including the effect of various parameters. We have tried to optimize lipase-catalyzed esterification and transesterification to ensure meaningful and objective comparison among different approaches. Synthesis of L-ascorbyl flurbiprofenate was influenced by reaction conditions. The equilibrium shift towards the L-ascorbyl flurbiprofenate synthesis was limited in spite of the presence of an excess of acyl donor. The most important strategy seemed to be the efficient removal of by-products, such as water or methanol. Addition of molecular sieves 3 Å during reaction could control water activity of the system. However, addition of molecular sieves 3 Å during reaction could not control methanol content. It was observed that the rate of the transesterification was much higher than that of esterification within 24 h. If the experiments were conducted for a long time, the conversion would reach asymptotic values and then the conversion of esterification was higher than that of the transesterification. This reflected a greater reactivity of flurbiprofen methyl ester in the lipase-catalyzed transesterification reaction as compared with esterification. However, the inherent drawback associated with lipase-catalyzed transesterification was the production of methanol and the equilibrium was usually not in favor of transesterification. Also, in the transesterification, flurbiprofen had to be chemically converted to its methyl ester and then subjected to the transesterification catalyzed by lipase. Although the rate of esterification is slower than that of transesterification, if a choice is at all available, from the standpoint of productivity and the amount of steps required, lipase-catalyzed esterification has been judged to be superior for the synthesis of L-ascorbyl flurbiprofenate.

## Figures and Tables

**Table 1:** The effect of solvent on the conversion of esterification and transesterification.

Solvent	log⁡*P*	Conversion of L-ascorbic acid (%)	
		Esterification	Transesterification
Acetone	−0.23	4.6	30.0
Tert-butanol(2-methyl-2-propanol)	0.60	36.0	38.5
Ethyl acetate	0.68	11.1	9.8
Tert-amyl alcohol; 2-methyl-butanol	0.89	35.3	36.5
Benzene	2.00	—	—
Chloroform	2.00	3.5	—
Toluene	2.50	—	—
